# Semi-Synthetic Cannabinoids in Forensic Toxicology and Public Health: Analytical Challenges, Emerging Detection Strategies, and Regulatory Implications

**DOI:** 10.3390/ph19071022

**Published:** 2026-06-30

**Authors:** Abdullah F. Aldasem, Sylvester N. Ugariogu, Abdullah Al-Matrouk, Naser F. Al-Tannak

**Affiliations:** 1 Saad Al-Abdullah Academy for Security Sciences, Shuwaikh Industial Area, Farwaniya Governorate, Kuwait City 70050, Kuwait; 2 Department of Pharmaceutical Chemistry, College of Pharmacy, Health Science Center, Kuwait University, P.O Box 24923, Jabriya 13110, Kuwait; 3 Quality Control Department, General Department of Forensic Science, Ministry of Interior, Farwaniya 85000, Kuwait

**Keywords:** semi-synthetic cannabinoids, hexahydrocannabinol, forensic toxicology, LC–MS/MS, high-resolution mass spectrometry, metabolite profiling, public health surveillance

## Abstract

Semi-synthetic cannabinoids (SSCs) are chemically modified derivatives of naturally occurring phytocannabinoids that have rapidly emerged in commercial cannabis and hemp-derived products, including vape cartridges, edibles, infused oils, and concentrated extracts. Increasing availability of compounds such as hexahydrocannabinol (HHC), HHC analogues, and Δ^8^-tetrahydrocannabinol (Δ^8^-THC) has created significant challenges for forensic toxicology, analytical detection, public health surveillance, and regulatory control. This structured narrative review evaluated current evidence on the forensic, toxicological, pharmacological, and analytical implications of SSCs. The literature published between January 2019 and May 2026 was identified through searches of PubMed, Scopus, and Web of Science using predefined search terms related to SSCs, forensic toxicology, analytical detection, intoxication, metabolism, and public health. Recent evidence demonstrates that HHC-related compounds currently dominate the SSC market and scientific literature. Available studies indicate that SSCs undergo extensive Phase I and Phase II metabolism, producing hydroxylated, oxidized, and glucuronidated metabolites that frequently predominate over parent compounds in biological matrices. This metabolic complexity complicates forensic interpretation, particularly in postmortem investigations and impairment assessments where toxicological reference ranges remain poorly established. Emerging intoxication reports describe prolonged sedation, neuropsychiatric manifestations, cognitive impairment, and severe poisoning associated with HHC analogues, although much of the current evidence remains limited to case reports and small observational studies. From an analytical perspective, conventional toxicology screening methods may fail to detect SSC exposure, necessitating advanced analytical approaches such as liquid chromatography–tandem mass spectrometry (LC–MS/MS), high-resolution mass spectrometry (HRMS), and chiral chromatographic techniques for metabolite identification and epimer differentiation. However, limited reference standards, evolving structural diversity, and regulatory variability across jurisdictions continue to hinder standardized detection and interpretation. Overall, SSCs represent a rapidly evolving class of psychoactive compounds requiring coordinated advancements in forensic toxicology, analytical surveillance, pharmacological characterization, and public health monitoring to improve detection reliability, risk assessment, and regulatory response.

## 1. Introduction

Semi-synthetic cannabinoids (SSCs) are chemically modified phytocannabinoid derivatives generated from naturally occurring cannabinoids, primarily cannabidiol (CBD) or Δ^9^-tetrahydrocannabinol (Δ^9^-THC), through chemical processes such as hydrogenation, acetylation, isomerization, or alkyl-chain modification [1,2,3]. Unlike naturally occurring phytocannabinoids isolated directly from *Cannabis sativa* or fully synthetic cannabinoids synthesized de novo, SSCs occupy an intermediate category because they originate from plant-derived cannabinoids that subsequently undergo laboratory-based structural modification [2,3]. This distinction is important in forensic and analytical toxicology because SSCs may demonstrate altered pharmacological activity, modified metabolic pathways, and unique analytical characteristics compared with both phytocannabinoids and synthetic cannabinoid receptor agonists (SCRAs) [3,4,5].

The emergence of SSCs has accelerated significantly in recent years, largely due to regulatory changes involving hemp-derived products. Following the 2018 United States Farm Bill, which legalized hemp products containing less than 0.3% Δ^9^-THC, cannabinoids such as Δ^8^-tetrahydrocannabinol (Δ^8^-THC) rapidly entered commercial markets as legal alternatives to cannabis products [6,7,8]. Since 2019, SSC-containing products, including vape cartridges, infused oils, edible gummies, tinctures, and concentrates marketed as “legal THC” alternatives, have become increasingly available through retail outlets and online platforms [7,9,10]. The rapid commercialization of these compounds, combined with inconsistent regulation and evolving production methods, has contributed to growing forensic, toxicological, and public health concerns [3,11].

Currently, the most prevalent SSCs include hexahydrocannabinol (HHC), hexahydrocannabiphorol (HHCP), HHC-O-acetate (HHC-O), tetrahydrocannabiphorol (THCP) analogues, and hydrogenated cannabidiol derivatives such as hexahydrocannabidiol (H4-CBD) [1,2,7]. HHC (6a,7,8,9,10,10a-hexahydro-6,6,9-trimethyl-3-pentyl-6H-dibenzo[b,d]pyran-1-ol) is among the most commonly detected SSCs and is generally synthesized through catalytic hydrogenation of Δ^8^-THC or Δ^9^-THC [4,12]. Although some SSCs may occur naturally in trace amounts, commercial products are predominantly manufactured through semi-synthetic conversion of hemp-derived CBD [13,14]. Recent forensic investigations have demonstrated increasing detection of SSCs in seized cannabis products and vaping formulations across Europe and North America [1,7,11].

The pharmacological properties of SSCs depend largely on their structural modifications and stereochemistry. HHC exists primarily as 9R- and 9S-epimers, with evidence suggesting that the 9R-isomer demonstrates greater affinity for cannabinoid receptor type 1 (CB1) and cannabinoid receptor type 2 (CB2) receptors [5,14]. In vitro studies have shown that HHC, HHC-O, and HHC-P exhibit cannabimimetic activity through CB1 receptor activation, although the potency and duration of effects may differ from conventional Δ^9^-THC [5]. Preliminary pharmacokinetic investigations additionally indicate prolonged psychophysical effects following oral and inhalational HHC exposure, although controlled human data remain limited.

Despite increasing recreational consumption, substantial gaps remain regarding the pharmacokinetics, toxicology, and long-term health consequences of SSC exposure. Most currently available evidence originates from analytical studies, poison centre reports, forensic case investigations, experimental metabolism studies, and isolated clinical case reports rather than large-scale epidemiological investigations [3,15,16]. Consequently, several proposed chronic risks remain speculative and should be interpreted cautiously. Nevertheless, reported adverse effects include sedation, agitation, anxiety, paranoia, psychosis, tachycardia, hypotension, bradycardia, impaired consciousness, and prolonged neurocognitive impairment [15,16,17]. Severe intoxication cases involving HHC, HHCP, and related analogues have also been documented following ingestion of edible formulations and vaping products [15,17].

The increasing market penetration of SSCs has generated significant public health concerns, particularly among adolescents and young adults attracted to products marketed as “legal,” “hemp-derived,” or safer alternatives to cannabis [8,9]. Several investigations have identified inconsistencies between product labelling and actual cannabinoid composition, with some commercial products containing undeclared SSCs, impurities, residual solvents, or additional psychoactive compounds [9,18,19]. Product variability is further complicated by the emergence of novel analogues and rapidly changing manufacturing practices [3,11]. User-reported experiences and online harm-reduction discussions additionally highlight concerns regarding mislabeled products, unpredictable psychoactive effects, and exposure to synthetic adulterants in unregulated markets.

From a forensic toxicology perspective, SSCs present substantial analytical and interpretative challenges. Many SSCs undergo extensive metabolism and produce structurally related metabolites that complicate toxicological interpretation [4,20,21]. Conventional immunoassays frequently fail to detect emerging SSC analogues, necessitating advanced chromatographic and mass spectrometric methods for reliable identification [3,22]. Analytical complexity is increased by the presence of positional isomers, stereoisomers, and epimers that may exhibit highly similar fragmentation patterns during tandem mass spectrometric analysis [3,14]. Consequently, advanced analytical platforms including liquid chromatography–tandem mass spectrometry (LC–MS/MS), high-resolution mass spectrometry (HRMS), gas chromatography–mass spectrometry (GC–MS), ultra-high-performance liquid chromatography coupled with quadrupole time-of-flight mass spectrometry (UHPLC-QTOF-MS), and chiral chromatographic separation methods are increasingly required for accurate SSC detection and differentiation [3,4,10,20,22,23,24].

An additional challenge involves legal and regulatory variability across jurisdictions. Some countries have explicitly controlled compounds such as HHC and Δ^8^-THC, whereas others regulate these substances indirectly through analogue legislation or broader psychoactive substance laws [3,11]. This inconsistency contributes to legal uncertainty regarding classification, forensic interpretation, toxicological reporting, and commercial distribution.

Although cannabinoids and synthetic cannabinoids have been extensively reviewed, relatively few studies have critically evaluated SSCs as a distinct and rapidly evolving subgroup characterized by unique analytical, toxicological, and public health implications [3]. Furthermore, much of the currently available evidence primarily concerns HHC-related compounds, representing an important limitation in the broader understanding of SSCs. Given the rapid expansion of SSC-containing products and the increasing number of intoxication reports, a comprehensive and critically structured review is necessary.

Therefore, this structured narrative review aims to evaluate the impact of SSCs on forensic toxicology and public health while critically examining emerging analytical detection strategies used for the identification, differentiation, and interpretation of SSCs and their metabolites. Particular emphasis is placed on forensic interpretation challenges, analytical limitations, public health implications, regulatory variability, and current evidence gaps relevant to toxicologists, forensic scientists, analytical chemists, and healthcare professionals.

**Figure 1:**
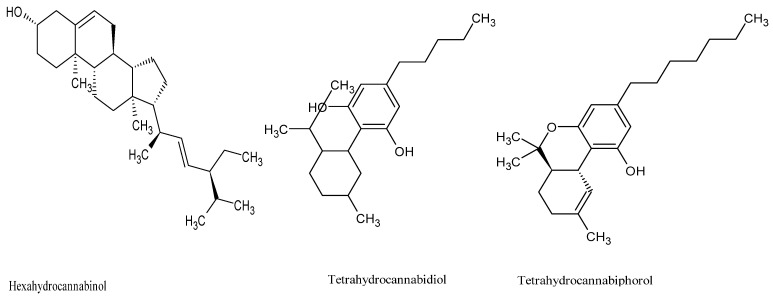
Structure of some semi-synthetic cannabinoids.

Representative structures of commonly encountered semi-synthetic cannabinoids are shown in Figure 1.

## 2. Methodology

### 2.1. Study Design

This study was conducted as a structured narrative review to synthesize current evidence regarding semi-synthetic cannabinoids (SSCs), with particular emphasis on forensic toxicology, public health implications, and advanced analytical detection techniques. The structured narrative approach was selected because the literature concerning SSCs remains rapidly evolving and highly heterogeneous, encompassing analytical validation studies, forensic case reports, toxicological investigations, pharmacological studies, poison centre reports, public health surveillance data, and regulatory publications [3,4,15].

Unlike a traditional narrative review, the structured narrative methodology incorporated predefined search strategies, eligibility criteria, thematic categorization, and structured data extraction procedures to improve methodological transparency and reproducibility. This approach was considered appropriate because the available evidence remains insufficiently standardized for quantitative meta-analysis and includes substantial methodological diversity across study types [3].

### 2.2. Literature Search Strategy

A comprehensive literature search was performed using the PubMed, Scopus, and Web of Science databases. The search covered studies published between January 2019 and June 2026 to capture recent developments in SSC emergence, metabolism, toxicology, analytical detection, and forensic interpretation [1,3].

The final database search was completed in June 2026. Boolean operators (“AND” and “OR”) were used to combine relevant search terms and keywords, including:

“semi-synthetic cannabinoids” “hexahydrocannabinol” OR “HHC” “hexahydrocannabiphorol” OR “HHCP” “HHC-O” OR “THC-O” “Δ^8^-THC” OR “Δ^10^-THC” “H4-CBD” “forensic toxicology” “public health” “clinical intoxication” “postmortem toxicology” “LC-MS/MS” “GC-MS” “HRMS” “UHPLC-QTOF-MS” “metabolites” “epimer differentiation” “isomer separation” “matrix effects” “analytical validation”

An example of the PubMed search syntax was:

(“semi-synthetic cannabinoids” OR HHC OR HHCP OR “Δ^8^-THC” OR “HHC-O”) AND (“forensic toxicology” OR “analytical detection” OR “public health” OR metabolites OR LC-MS/MS OR HRMS).

Additional studies were identified manually through reference-list screening of relevant reviews and primary analytical investigations [3,4].

### 2.3. Study Selection

All identified records were screened for duplicates, and duplicate studies were removed prior to screening. Two independent reviewers screened article titles and abstracts according to predefined inclusion and exclusion criteria. Potentially eligible studies subsequently underwent full-text assessment.

Disagreements during study selection and data extraction were resolved through discussion and consensus. Where disagreement persisted, a third reviewer was consulted to achieve final agreement.

The initial database search identified 142 records. After duplicate removal, 92 studies underwent title and abstract screening. A total of 65 articles were excluded because they lacked SSC relevance, focused exclusively on natural or fully synthetic cannabinoids, lacked methodological detail, or did not provide accessible full-text data. Subsequently, 27 full-text articles were evaluated for eligibility, and 25 studies were included in the final qualitative synthesis.

### 2.4. Inclusion and Exclusion Criteria

#### 2.4.1. Inclusion Criteria

Studies were included if they met one or more of the following criteria:

Peer-reviewed journal articles published in English; studies involving SSCs in forensic toxicology, analytical chemistry, clinical toxicology, or public health contexts; research investigating analytical techniques, including LC–MS/MS, GC–MS, HRMS, UHPLC-QTOF-MS, or chiral chromatographic separation methods [4,10,23,24]; studies evaluating SSC metabolism, biomarkers, pharmacology, intoxication, or toxicological interpretation [5,14,21,22]; experimental investigations, case reports, surveillance studies, analytical validation studies, and forensic case series.

#### 2.4.2. Exclusion Criteria

Studies were excluded if they met any of the following criteria:

Non-English publications; conference abstracts without accessible full-text articles; studies focused exclusively on naturally occurring cannabinoids or fully synthetic cannabinoids without direct SSC relevance; editorials, commentaries, and opinion articles lacking primary analytical or toxicological data; studies with insufficient methodological rigour or inadequate analytical detail.

### 2.5. Data Extraction and Thematic Synthesis

Data extraction was independently performed by two reviewers using a standardized extraction framework. The following information was collected from eligible studies:

Publication year and study design; SSC compounds investigated; biological and forensic matrices analyzed; analytical instrumentation and detection methods; target analytes and metabolites identified; validation parameters including extraction recovery, matrix effects, sensitivity, specificity, linearity, and limits of detection where available [4,10,24,25]; toxicological and pharmacological findings; clinical and forensic implications; public health and regulatory considerations; limitations relevant to forensic interpretation.

Extracted findings were categorized into the following thematic areas:

- Emergence and market distribution of SSCs.
- Pharmacology, metabolism, and toxicological effects.
- Clinical intoxication and forensic case implications.
- Public health concerns and vulnerable populations.
- Postmortem interpretation challenges.
- Legal and regulatory implications.
- Advanced analytical detection techniques and analytical limitations.

A qualitative synthesis approach was used to identify recurring themes, analytical challenges, methodological strengths, limitations, and evidence gaps. Particular emphasis was placed on issues involving metabolite-focused analysis, epimer and isomer differentiation, matrix effects, extraction recovery, chiral separation, and the limitations of routine toxicological screening approaches [3,4,14,20,21,22,23,24].

### 2.6. Quality Appraisal and Limitations

Because this review included heterogeneous evidence sources such as case reports, analytical validation studies, surveillance investigations, and experimental pharmacological studies, a formal meta-analysis and quantitative risk-of-bias assessment were not performed.

Nevertheless, methodological quality was critically evaluated during study selection. Greater emphasis was placed on studies with validated analytical methodologies, clearly described experimental procedures, adequate analytical specificity, and well-documented forensic or clinical findings [4,10,20,21].

Several limitations should be acknowledged. First, much of the currently available evidence concerns HHC-related compounds, limiting broader generalization to all SSC subclasses [3]. Second, the rapid evolution of the SSC market means newly emerging compounds may not yet be adequately represented in the scientific literature. Third, several toxicological conclusions currently rely on isolated case reports, poison centre data, or preliminary experimental studies rather than large-scale epidemiological investigations [3,15,16]. Consequently, distinctions were made throughout this review between established findings, plausible risks, and areas where evidence remains limited.

### 2.7. Ethical Considerations

Ethical approval and informed consent were not required because this review was based exclusively on the previously published literature and did not involve human participants, animal experimentation, or access to confidential patient information.

## 3. Impact of Semi-Synthetic Cannabinoids on Forensic Toxicology

Semi-synthetic cannabinoids (SSCs) have emerged as a major analytical, toxicological, and regulatory concern in forensic science due to their structural diversity, rapid market expansion, and evolving pharmacological profiles. Unlike naturally occurring phytocannabinoids, SSCs are chemically modified cannabinoid derivatives generated through processes such as hydrogenation, acetylation, or isomerization of cannabidiol (CBD) and Δ^9^-tetrahydrocannabinol (Δ^9^-THC). The growing commercial availability of compounds such as hexahydrocannabinol (HHC), hexahydrocannabinabiphorol (HHCP), HHC-O-acetate, Δ^8^-THC, and Δ^10^-THC has significantly complicated forensic toxicology, particularly in relation to analytical detection, metabolite interpretation, impairment assessment, and postmortem investigations [1,3,4,7].

### 3.1. Emergence, Prevalence, and Market Complexity

Recent seizure analyses and market surveillance studies demonstrate a substantial increase in the prevalence of SSC-containing products across Europe and North America [1,7,11]. HHC derivatives, Δ^8^-THC products, and acetylated cannabinoids are increasingly identified in vape cartridges, gummies, infused oils, and edible formulations marketed as “legal” or “hemp-derived” alternatives to cannabis [7,8,9,19]. The expansion of these products accelerated after the legalization of hemp-derived CBD products in several jurisdictions, particularly following the 2018 United States Farm Bill [8].

Forensic investigations have shown that commercial SSC products frequently contain inaccurate cannabinoid labelling, undeclared psychoactive compounds, residual synthesis by-products, and inconsistent cannabinoid concentrations [9,18,19]. Product profiling studies identified substantial variability in potency and composition between products marketed under similar names, creating significant toxicological and public health concerns [7,18]. Such inconsistencies complicate forensic interpretation because exposure may involve multiple cannabinoids, impurities, or unidentified analogues rather than a single compound [9,11].

Regional differences in SSC prevalence have also been reported. Seizure studies from Denmark, Germany, and Poland indicate increasing circulation of HHC analogues and acetylated cannabinoids in recreational drug markets [1,7,11]. Younger individuals and recreational users appear to be disproportionately represented among reported intoxication cases, particularly through vaping products and edible formulations [10]. The widespread online availability of these products further complicates surveillance efforts and contributes to the rapid dissemination of new SSC analogues before regulatory control measures can be implemented [3].

### 3.2. Analytical Challenges of Detection and Identification

The analytical identification of SSCs presents substantial challenges due to structural similarity among cannabinoids, the presence of positional and stereoisomers, and the rapid emergence of novel analogues [3,4]. Routine toxicological screening methods, including conventional immunoassays and standard cannabis testing panels, frequently fail to detect SSC exposure because many compounds are absent from existing analytical libraries and reference databases [16,22,23].

Several SSCs possess highly similar fragmentation patterns and overlapping chromatographic characteristics, increasing the risk of misidentification or false-negative findings [3,9]. Analytical differentiation becomes particularly difficult for structurally related compounds such as HHC epimers and Δ^8^-/Δ^9^-THC analogues [14,22]. The absence of certified reference standards for newly emerging compounds further compromises confirmatory analysis, quantitative reliability, and legal defensibility in forensic casework [7].

Moreover, SSC detection is complicated by matrix effects, thermal instability, and limited chromatographic resolution during routine screening [4,25]. Traditional gas chromatography-mass spectrometry (GC-MS) methods may induce thermal degradation or structural conversion of thermolabile SSCs, necessitating derivatization procedures that increase analytical uncertainty [11]. Consequently, forensic laboratories increasingly rely on advanced liquid chromatography-based methods combined with tandem or high-resolution mass spectrometry to improve analytical specificity and sensitivity [3,4,23].

### 3.3. Metabolic Complexity and Biomarker Identification

SSCs undergo extensive phase I and phase II biotransformation, producing complex metabolic profiles consisting of hydroxylated, oxidized, carboxylated, and glucuronidated metabolites [4,20,21]. In many biological matrices, parent compounds are either absent or present only at trace concentrations, particularly in urine samples where glucuronide conjugates dominate [14,21,22]. As a result, metabolite-focused analysis has become essential for reliable confirmation of SSC exposure.

Recent studies demonstrated that HHC metabolism produces multiple metabolites, including 11-hydroxy-HHC and HHC-COOH derivatives, many of which overlap structurally with Δ^9^-THC metabolites [22,23]. Such overlap complicates forensic interpretation because conventional cannabinoid assays may not adequately distinguish SSC metabolites from those derived from traditional cannabis consumption [23]. Furthermore, epimer-specific metabolism contributes additional complexity. The 9(R)- and 9(S)-HHC stereoisomers exhibit different metabolic pathways, receptor affinities, and biomarker distributions [5,14].

High-resolution analytical approaches have enabled the identification of epimer-specific biomarkers and previously unknown metabolites in authentic urine and blood samples [14,20]. Nevertheless, an incomplete understanding of SSC pharmacokinetics and metabolite persistence continues to limit interpretation regarding the timing of intake, extent of exposure, and impairment assessment [3].

### 3.4. Toxicological and Pharmacological Uncertainty

The toxicological interpretation of SSC exposure remains challenging because pharmacodynamic and pharmacokinetic data are still limited for many emerging analogues [3,12]. Although several SSCs act as partial agonists of cannabinoid receptors, particularly CB1 receptors, receptor affinity, potency, and psychoactive effects vary considerably depending on structural modifications and stereochemistry [2,5].

Experimental studies demonstrated that HHC derivatives and acetylated cannabinoids may exhibit stronger or more prolonged cannabimimetic activity than naturally occurring Δ^9^-THC [5,12]. The pharmacological activity of HHC is especially dependent on stereochemistry, with the 9(R)-epimer generally displaying greater CB1 receptor activation than the 9(S)-epimer [5,14]. Such variability complicates forensic interpretation because identical measured concentrations may not correspond to equivalent levels of intoxication or impairment.

Current evidence regarding chronic toxicity, neurotoxicity, dependency potential, and long-term psychiatric effects remains limited. Most available data originate from case reports, in vitro receptor studies, or extrapolation from the synthetic cannabinoid literature [3]. Therefore, it is important to distinguish between established toxicological findings and plausible but insufficiently validated risks.

### 3.5. Clinical and Forensic Case Implications

Emerging clinical reports demonstrate that SSC intoxication can produce severe and prolonged toxicological manifestations, including sedation, unconsciousness, seizures, cognitive impairment, psychomotor dysfunction, hypotension, bradycardia, and psychiatric symptoms [15,16,17,24]. Several reported cases involved products marketed as legal cannabis alternatives, emphasizing the risks associated with product mislabeling and uncontrolled potency [9,18].

Severe poisoning cases involving HHC-C8 and HHC-C9 analogues demonstrated prolonged neurological impairment and delayed recovery despite initially negative routine toxicology screens [16,17]. Controlled pharmacokinetic investigations further indicated that HHC exposure may produce persistent psychophysical impairment following both oral and inhalational administration [18].

Forensic interpretation of these cases remains difficult because clinical symptoms may differ substantially from classical cannabis intoxication and because exposure often involves multiple cannabinoids or impurities [3,7]. In many cases, advanced LC-MS/MS or HRMS techniques were required to confirm SSC exposure after standard toxicological testing failed to detect the responsible compounds [16,23].

### 3.6. Postmortem Interpretation and Forensic Uncertainty

Postmortem toxicological interpretation involving SSCs remains highly challenging because of limited reference concentration data, incomplete pharmacokinetic knowledge, and substantial metabolite overlap with conventional cannabinoids [3,4]. Parent compounds may be absent in postmortem matrices due to extensive metabolism, instability, or redistribution, while metabolites often persist at detectable concentrations [21,22].

The absence of established toxic, impairing, or fatal concentration ranges further limits interpretation of the cause of death or contribution to impairment [11]. Additionally, postmortem cases frequently involve polydrug exposure, complicating attribution of toxicological effects to a single SSC [3]. Studies involving HHC metabolites demonstrated that some biomarkers overlap with Δ^9^-THC metabolites, increasing the potential for interpretive ambiguity during forensic investigations [22,23].

Consequently, forensic experts must interpret SSC findings cautiously and within the broader context of clinical history, scene evidence, autopsy findings, and co-occurring substances.

### 3.7. Legal and Regulatory Implications

The rapid emergence of SSCs has created substantial legal and regulatory uncertainty across multiple jurisdictions because many of these compounds are synthesized from legally cultivated hemp-derived cannabidiol (CBD) while producing psychoactive effects comparable to Δ^9^-THC [3,8,11]. Manufacturers frequently exploit legislative gaps by introducing structurally modified cannabinoids that are not yet explicitly listed within national controlled substance schedules [3,7]. This has resulted in a continuously evolving market characterized by rapid chemical diversification and inconsistent regulatory oversight.

Regulatory approaches toward SSCs vary considerably between countries and regions. Several European jurisdictions, including France and Austria, have implemented direct restrictions on compounds such as HHC and related analogues following reports of severe intoxications and increasing forensic detections [3,4]. In contrast, other countries regulate SSCs indirectly through analogue legislation, generic scheduling systems, or broader psychoactive substance laws, creating variability in enforcement and legal interpretation [3,11]. In the United States, legal ambiguities associated with the 2018 Farm Bill and hemp-derived CBD have contributed to the widespread commercial availability of Δ^8^-THC, HHC, and related SSC products marketed as “legal THC” alternatives [8].

This regulatory inconsistency complicates forensic toxicology practice in several ways. First, forensic laboratories must continuously update analytical libraries and validated screening methods to accommodate newly emerging SSC analogues [3,7]. Second, toxicologists and legal experts may face challenges in determining the legal status of confiscated substances when compounds are not specifically listed in legislation [11]. Third, variations in product composition, inaccurate labelling, and the presence of multiple SSC analogues within single products complicate toxicological interpretation and courtroom testimony [9,18,19].

Another important concern is the emergence of rapidly modified SSC analogues designed specifically to circumvent existing drug control laws [7]. Structural alterations such as hydrogenation, acetylation, alkyl-chain modification, and epimeric variation may produce compounds with altered pharmacological properties while remaining outside existing legal definitions [2,3]. Consequently, several authors have advocated for broader structure-based or generic scheduling frameworks rather than compound-specific legislation to improve regulatory responsiveness [3].

From a forensic and public health perspective, harmonized international regulation and standardized reporting systems are urgently needed. Coordinated early warning systems, inter-laboratory collaboration, and improved communication between forensic scientists, toxicologists, regulatory agencies, and healthcare professionals are essential for effective surveillance and rapid identification of emerging SSC threats [3,7]. Such integrated approaches may improve public safety, support forensic defensibility, and reduce delays in regulatory intervention associated with newly emerging semi-synthetic cannabinoids.

### 3.8. Implications for Forensic Practice

The rapid emergence of SSCs has significantly altered contemporary forensic toxicology workflows. Traditional cannabinoid screening strategies that primarily target Δ^9^-THC and its metabolites are increasingly insufficient for detecting newer SSC analogues, particularly HHC derivatives and acetylated compounds [3,4,7]. Several forensic investigations demonstrated that routine toxicology panels may fail to detect SSC exposure, particularly when laboratories rely exclusively on conventional immunoassays or outdated spectral libraries [4,16,17].

Consequently, forensic laboratories are transitioning toward broader analytical workflows that integrate targeted LC–MS/MS screening with non-targeted HRMS-based approaches [3,7]. Metabolite-focused interpretation has become especially important because parent compounds are frequently absent or present at very low concentrations in biological matrices such as urine and postmortem blood [14,20,21,22,23]. This shift requires continuous updating of analytical libraries, acquisition of certified reference standards, and implementation of validated multi-platform workflows capable of resolving structural isomers and epimers [7,11].

The increasing complexity of SSC-related casework also has implications for forensic interpretation. Toxicologists are often required to provide expert opinions despite limited pharmacokinetic data, uncertain toxic concentration ranges, and overlapping metabolic profiles with Δ^9^-THC [3,4,14]. These uncertainties complicate impairment assessment, postmortem interpretation, and medicolegal decision-making. Therefore, harmonized analytical guidelines, inter-laboratory collaboration, and standardized reporting frameworks are urgently needed to improve consistency and legal defensibility in SSC investigations [3,26].

## 4. Impact of Semi-Synthetic Cannabinoids on Public Health

### 4.1. Increasing Availability, Product Diversification, and Exposure Risks

SSCs are increasingly marketed through commercial products including vape cartridges, gummies, infused oils, tinctures, chocolates, and concentrated “legal THC” formulations [7,8,9,10]. Products marketed as Δ^8^-THC or HHC formulations have expanded rapidly in both online and retail markets following legal loopholes associated with hemp-derived cannabidiol (CBD) [8,18]. Epidemiological seizure data from Denmark, Germany, and Poland indicate a substantial increase in the prevalence and diversity of SSC-containing products between 2019 and 2025 [1,7,11].

Multiple analytical investigations identified inaccurate labelling, inconsistent cannabinoid concentrations, undeclared psychoactive compounds, and contamination with synthetic byproducts generated during chemical conversion processes [9,18,19]. Acid-catalyzed conversion of CBD to Δ^8^-THC or HHC may produce numerous unidentified impurities and reaction byproducts with uncertain toxicological properties [18,19]. Such variability increases the risk of accidental overexposure, unpredictable intoxication, and adverse health outcomes [20].

Commercial promotion of SSC products as “legal,” “natural,” or “safer” alternatives to cannabis may further contribute to consumer misconceptions regarding safety and potency [20]. These concerns are particularly important because many products bypass pharmaceutical-quality manufacturing controls and standardized labelling requirements [7,9].

### 4.2. Acute Toxicity and Clinical Manifestations

Emerging clinical evidence indicates that SSC exposure can produce a broad spectrum of adverse effects ranging from mild intoxication to severe neuropsychiatric and cardiovascular complications [15,16,17]. Reported manifestations include sedation, agitation, anxiety, confusion, hallucinations, impaired consciousness, seizures, bradycardia, hypotension, tachycardia, and prolonged cognitive dysfunction [15,16,17].

Severe intoxication cases involving HHC, HHC-P, HHC-C8, and HHC-C9 have been documented in both the emergency medicine and forensic literature [15,16,17]. Thomsen et al. [17] reported prolonged unconsciousness and sedation following HHC-C8 exposure, while Reiter et al. [16] described persistent neurological and cognitive impairment after recreational use of HHC-C9. Importantly, several cases initially produced negative routine toxicology findings and required advanced LC–MS/MS or HRMS analysis for definitive identification [16,17].

Current evidence remains limited primarily to case reports and small clinical datasets. Therefore, many proposed long-term risks remain hypothetical or extrapolated from synthetic cannabinoid research rather than directly established for SSCs [3]. Clear distinctions between demonstrated toxic effects and plausible risks should therefore be maintained.

### 4.3. Vulnerable Populations and Exposure Patterns

Available evidence suggests that adolescents, young adults, pregnant women, and neonates may represent particularly vulnerable populations for SSC exposure [8,10,13]. Youth exposure is strongly associated with vaping products, flavoured edibles, and commercially marketed “hemp-derived THC” products [8,10]. The attractive packaging, flavouring, and perceived legal status of these products may contribute to increased experimentation among younger users [8].

Ballotari et al. [13] demonstrated the presence of cannabinoids and SSCs in authentic breastmilk samples using validated LC–MS/MS methods, raising concerns regarding neonatal exposure during breastfeeding. Although direct clinical consequences remain insufficiently characterized, neonatal cannabinoid exposure may present developmental and neurological concerns that warrant further investigation [13].

Patterns of exposure also differ between recreational consumers and individuals unintentionally exposed through mislabeled commercial products [9,18]. Recreational users may intentionally seek highly potent SSC formulations, whereas other consumers may unknowingly ingest SSCs through inaccurately labelled CBD or hemp-derived products [9].

### 4.4. Public Health Surveillance and Epidemiological Challenges

Public health surveillance of SSCs remains difficult because of rapid market evolution, inconsistent analytical detection, and underreporting [3,7]. Seizure analyses from European forensic laboratories demonstrate substantial year-to-year changes in the composition and prevalence of SSC-containing products [1,7,11]. However, true population prevalence remains uncertain because many standard toxicology programmes do not routinely screen for SSCs [3].

Analytical under-recognition is further complicated by extensive metabolism and the predominance of metabolites over parent compounds in biological samples [14,20,21,22]. Conventional screening programmes may therefore underestimate SSC exposure prevalence in both clinical and forensic populations [4].

Early warning systems and poison centre monitoring programmes play increasingly important roles in identifying emerging SSC-related harms [3]. Integration of forensic toxicology data, poison centre reports, emergency department presentations, and seizure analyses may improve real-time epidemiological surveillance and facilitate earlier public health interventions [3,7].

### 4.5. Regulatory and Policy Challenges

The legal status of SSCs varies substantially across jurisdictions, contributing to regulatory uncertainty and inconsistent enforcement [3,11]. Some countries have implemented direct controls targeting compounds such as Δ^8^-THC, HHC, and HHC derivatives, whereas others regulate these substances indirectly under analogue legislation or broader psychoactive substance frameworks [3].

European countries, including France and Austria, have introduced restrictions on HHC-related compounds following reports of intoxication and increasing market prevalence [3,4]. In contrast, other jurisdictions continue to permit the commercial sale of hemp-derived SSC products due to legal ambiguities surrounding CBD conversion processes [8]. These inconsistencies complicate forensic classification, cross-border regulation, and public health policy implementation [3,11].

The rapid structural modification of SSCs also enables manufacturers to circumvent legislation through the introduction of newly modified analogues [3,7]. Consequently, several authors have advocated for broader structure-based or generic scheduling frameworks rather than compound-specific regulation [3].

### 4.6. Risk Communication and Clinical Preparedness

Healthcare systems increasingly face diagnostic and management challenges associated with SSC intoxication [16,17]. Clinical presentation may differ substantially from traditional cannabis intoxication, and routine toxicology screening frequently fails to identify exposure [4,16]. This may delay diagnosis, compromise patient management, and hinder accurate epidemiological reporting.

Improved clinician awareness, updated toxicology testing protocols, and expanded access to advanced analytical testing are therefore essential [3]. Public health communication strategies should also emphasize the risks associated with mislabeled and unregulated SSC products, particularly among adolescents, pregnant women, and inexperienced consumers [8,9,13].

Risk communication should avoid overstating evidence where data remain limited. Current knowledge regarding chronic toxicity, neurodevelopmental consequences, dependence potential, and long-term psychiatric effects remains incomplete and requires further investigation [3].

The principal findings, analytical approaches, forensic implications, and public health relevance of the reviewed studies are summarized in Table 1 below.

A descriptive synthesis of the included studies demonstrated that hexahydrocannabinol (HHC) and related analogues remain the most extensively investigated SSCs, particularly in forensic toxicology and analytical chemistry applications. Recent studies increasingly emphasize metabolite-centred toxicology, epimer-specific interpretation, and advanced LC–MS/MS or HRMS workflows for detecting novel SSC analogues and biomarkers [4,14,20,21,23]. Product surveillance investigations consistently reported widespread mislabeling, undeclared cannabinoid additives, and variable potency in commercial vaping liquids and edible formulations [9,10,18,19]. Clinical reports further demonstrated severe intoxication outcomes, including prolonged unconsciousness, neurological impairment, and persistent cognitive dysfunction following HHC analogue exposure [15,16,17]. Collectively, the reviewed evidence highlights the urgent need for harmonized analytical frameworks, improved forensic surveillance systems, updated regulatory approaches, and expanded pharmacotoxicological investigations to address the rapidly evolving SSC landscape.

## 5. Advanced Analytical Detection Strategies Used in the Detection of Semi-Synthetic Cannabinoids

### 5.1. Limitations of Conventional Screening Approaches

Conventional immunoassays and standard cannabinoid testing panels are often inadequate for SSC detection because many emerging compounds demonstrate limited cross-reactivity with traditional assays [3,4]. As a result, SSC exposure may remain undetected during routine toxicological analysis [16,17].

Traditional GC–MS methods also present analytical limitations because several SSCs exhibit thermal instability and poor volatility, requiring derivatization prior to analysis [7,11]. Thermal degradation during GC analysis may further complicate interpretation and reduce analytical reliability [11]. These limitations have accelerated the adoption of LC-based and HRMS-based analytical workflows for SSC detection.

### 5.2. LC–MS/MS as the Core Targeted Analytical Platform

LC–MS/MS remains the primary analytical technique for targeted SSC detection because of its high sensitivity, selectivity, and suitability for thermolabile compounds [3,6,7,19]. Validated LC–MS/MS methods have successfully quantified SSCs and metabolites in blood, urine, breastmilk, vaping liquids, and edible products [9,13,23].

Most methods employ reverse-phase chromatography with C18 columns combined with electrospray ionization in positive ionization mode and multiple reaction monitoring (MRM) acquisition [6,19]. These workflows provide low limits of detection, acceptable extraction recovery, high precision, and broad calibration ranges appropriate for forensic and clinical applications [9,13].

However, LC–MS/MS methods may demonstrate limited capability for distinguishing positional isomers and stereoisomers that generate similar fragmentation patterns [11,14]. Consequently, complementary HRMS or chiral chromatographic approaches are increasingly required for definitive compound identification.

### 5.3. High-Resolution Mass Spectrometry and Non-Targeted Screening

HRMS platforms, including Orbitrap and quadrupole time-of-flight (QTOF) instruments, have become essential for non-targeted SSC screening and metabolite characterization [3,4,7]. HRMS enables accurate mass determination, retrospective data analysis, structural elucidation, and detection of previously unknown analogues [7].

Several studies used LC-HRMS and UHPLC-QTOF-MS to characterize HHC metabolites, identify novel SSC analogues, and establish biomarker profiles in authentic biological samples [4,14,20,21]. Untargeted metabolomics workflows are particularly valuable because SSC markets evolve rapidly and newly emerging compounds may not yet exist in targeted analytical libraries [3].

Despite these advantages, HRMS workflows may still face challenges related to stereoisomer differentiation, data interpretation complexity, and the requirement for specialized expertise [3,11].

### 5.4. Isomer and Epimer Differentiation

Differentiation of SSC isomers, stereoisomers, and epimers represents one of the most important unresolved analytical challenges in forensic toxicology [11,14]. Many SSC analogues generate highly similar fragmentation spectra and overlapping chromatographic behaviour, limiting the discriminatory power of conventional MRM-based LC–MS/MS methods [14].

Epimer-specific metabolism of HHC further complicates interpretation because 9(R)-HHC and 9(S)-HHC exhibit distinct pharmacological activity and metabolic profiles [5,14]. Gameli et al. [14] identified diagnostic biomarkers capable of distinguishing exposure to individual HHC epimers, highlighting the importance of stereoselective analysis.

Advanced chromatographic approaches, including chiral chromatography, improved stationary phase selection, and optimized gradient conditions, may improve stereoisomer separation [11,14]. HRMS fragmentation analysis also contributes additional structural information that may support differentiation of closely related analogues [3].

### 5.5. Multi-Platform Analytical Strategies and Method Validation

No single analytical technique is currently sufficient for comprehensive SSC detection. Consequently, multi-platform workflows integrating LC–MS/MS, HRMS, GC–MS, and metabolomics approaches are increasingly recommended [3,7].

Method validation remains essential for ensuring forensic reliability and legal defensibility [26]. Key validation parameters include selectivity, extraction recovery, matrix effects, accuracy, precision, calibration linearity, carryover assessment, and limits of detection and quantification [9,13,25]. Matrix effects are particularly important when analyzing complex biological specimens such as blood, urine, breastmilk, and postmortem tissues [13,25].

Continuous updating of spectral libraries and reference standards is also necessary because newly emerging SSC analogues rapidly outpace existing forensic databases [3,7].

A comparative overview of the principal analytical platforms currently used for SSC detection, metabolite characterization, and forensic interpretation is presented in Table 2 below.

Table 2 highlights the major analytical techniques currently used for the detection of semi-synthetic cannabinoids (SSCs). LC–MS/MS remains the primary method because of its high sensitivity, selectivity, and suitability for quantitative analysis in biological samples [4,9,13]. HRMS techniques such as LC-QTOF-MS and Orbitrap provide important advantages for non-targeted screening, metabolite profiling, and identification of newly emerging SSC analogues [3,7,20,21].

Although GC–MS is still widely used for seized material analysis, its application is limited by the thermal instability and derivatization requirements of several SSCs [11,17]. In addition, metabolomics and UHPLC-based approaches have improved the identification of hydroxylated, oxidized, and glucuronidated metabolites that often predominate in biological matrices [14,20,21].

Overall, the reviewed studies demonstrate that integrated multi-platform analytical strategies are necessary for reliable SSC detection, accurate metabolite identification, and differentiation of structurally similar isomers and epimers.

## 6. Research Gaps and Limitations

Despite the growing number of studies on semi-synthetic cannabinoids (SSCs), major scientific, analytical, toxicological, and regulatory gaps remain. One of the most important limitations identified in the reviewed literature is the lack of comprehensive human pharmacokinetic and pharmacodynamic data for compounds such as hexahydrocannabinol (HHC), HHC-P, HHC-O, HHC-C8, and related analogues [4,12,14,20,21]. Most available evidence is derived from in vitro metabolism studies, seized-product investigations, analytical validation studies, or isolated clinical case reports rather than controlled human investigations. Consequently, reliable concentration–effect relationships, impairment thresholds, toxic ranges, and fatal concentrations remain poorly established, thereby limiting forensic interpretation and medico-legal decision-making.

Another major limitation is the rapid structural evolution of SSCs, which frequently outpaces current analytical libraries, certified reference standards, and legislative scheduling systems [1,3,7,11]. Newly emerging SSC analogues may evade routine toxicological screening because conventional immunoassays and standard cannabis panels often fail to detect structurally modified cannabinoids or their metabolites [4,16,17]. Although advanced LC–MS/MS and HRMS workflows improve detection capability, inter-laboratory harmonization remains limited, and validated analytical protocols for many novel SSCs are still unavailable.

The differentiation of positional isomers, stereoisomers, and epimers also remains a substantial analytical challenge. Recent studies demonstrated that 9(R)- and 9(S)-HHC epimers exhibit different metabolic profiles and CB1 receptor activities, which may significantly influence toxicological interpretation [5,14]. However, validated chiral chromatographic approaches and epimer-specific reference standards are not yet widely implemented in routine forensic laboratories. This analytical limitation may result in inaccurate substance attribution, inconsistent reporting, and reduced forensic defensibility.

Public health surveillance is similarly constrained by insufficient epidemiological data and under-recognition of SSC exposure. Product mislabeling, inconsistent potency, and undeclared cannabinoid additives remain common in commercial vaping liquids, edibles, and hemp-derived products [9,10,18,19]. Nevertheless, population-level prevalence studies, poison centre analyses, longitudinal health assessments, and standardized exposure monitoring systems remain limited across many regions. Current evidence is therefore heavily biased toward acute intoxication and forensic casework rather than long-term health outcomes.

Additional limitations include the predominance of small sample sizes, regionalized seizure analyses, and reliance on retrospective or descriptive methodologies. Many studies focus primarily on HHC and related analogues, while alternative SSC scaffolds remain poorly characterized. Furthermore, limited toxicological data on chronic exposure, neurocognitive consequences, psychiatric complications, cardiovascular toxicity, reproductive effects, and dependence potential restricts comprehensive risk assessment. Regulatory fragmentation across jurisdictions further complicates forensic classification, public health response, and international monitoring efforts.

The reviewed literature demonstrates that SSC research remains an evolving field characterized by methodological fragmentation, limited standardization, and substantial toxicological uncertainty. Coordinated multidisciplinary investigations integrating forensic toxicology, analytical chemistry, pharmacology, epidemiology, and public health surveillance are therefore urgently needed to improve scientific understanding and support evidence-based regulatory strategies.

## 7. Future Perspectives

Future research on semi-synthetic cannabinoids should prioritize the development of comprehensive human pharmacokinetic and pharmacodynamic datasets capable of establishing reliable relationships between dose, blood concentration, impairment, toxicity, and clinical outcomes. Controlled clinical and toxicological studies are necessary to define absorption, distribution, metabolism, elimination, and concentration–effect relationships for emerging SSCs, particularly HHC analogues and newly synthesized derivatives [12,14,20,21]. Such information would significantly improve forensic interpretation, postmortem toxicology, roadside impairment investigations, and medico-legal assessments.

Analytically, future forensic workflows should increasingly adopt metabolite-focused and epimer-specific detection strategies supported by advanced LC–MS/MS, HRMS, and chiral chromatographic methodologies. The continued expansion of spectral libraries, validated reference standards, and harmonized analytical protocols will be essential for improving inter-laboratory reproducibility and detection reliability [3,7,27]. Integration of targeted and untargeted analytical approaches may further enhance the identification of newly emerging SSC analogues and previously uncharacterized metabolites in biological and seized materials.

The implementation of coordinated international early warning systems and integrated surveillance networks will also be critical for addressing the rapidly evolving SSC market. Combining forensic seizure data, poison centre reports, emergency department observations, toxicological findings, and public health monitoring may facilitate earlier recognition of harmful compounds and improve risk assessment capabilities. Greater collaboration between forensic laboratories, healthcare institutions, toxicologists, public health agencies, and regulatory authorities is therefore strongly recommended.

From a public health perspective, future investigations should focus on vulnerable populations, including adolescents, pregnant women, breastfeeding mothers, and individuals with underlying psychiatric or cardiovascular disorders. Longitudinal studies evaluating neurocognitive impairment, dependency potential, chronic toxicity, reproductive outcomes, and psychiatric sequelae remain urgently needed. Improved consumer education, standardized labelling regulations, and harm-reduction initiatives may additionally reduce accidental exposure and product misuse.

Regulatory frameworks may also require a transition from compound-specific scheduling toward broader structure-based or analogue-based legislative approaches capable of adapting to rapid chemical modifications. International harmonization of legal classifications, analytical reporting criteria, and toxicological guidelines would strengthen forensic consistency and improve global surveillance efforts. Ultimately, proactive multidisciplinary collaboration integrating analytical toxicology, pharmacology, clinical medicine, epidemiology, and public health policy will be essential for addressing the continuing emergence of SSCs and mitigating associated forensic and societal risks.

## 8. Conclusions

Semi-synthetic cannabinoids represent a rapidly expanding and analytically challenging class of emerging psychoactive substances with significant implications for forensic toxicology, public health, and regulatory policy. The reviewed literature demonstrates that compounds such as HHC and its analogues possess complex metabolic profiles characterized by extensive Phase I and II biotransformation, resulting in hydroxylated, oxidized, and glucuronidated metabolites that frequently predominate in biological specimens [4,14,20,21]. Consequently, metabolite-centred and epimer-specific analytical approaches are increasingly necessary for accurate toxicological interpretation.

Recent forensic and clinical investigations have linked SSC exposure to severe intoxication, prolonged sedation, neurological impairment, and persistent cognitive dysfunction, while routine toxicology panels often fail to detect these compounds [15,16,17]. The widespread availability of mislabeled or adulterated commercial products further increases exposure risk and complicates forensic attribution [9,18,19]. Simultaneously, rapid structural diversification and inconsistent international regulation continue to create major analytical and legal challenges.

Advanced analytical platforms, particularly LC–MS/MS and HRMS-based methodologies, currently represent the most effective tools for SSC detection, metabolite profiling, and identification of newly emerging analogues [3,4,20,21,27]. However, analytical harmonization, validated reference materials, chiral separation strategies, and comprehensive toxicological datasets remain insufficiently developed. The findings of this review therefore emphasize the urgent need for integrated multi-platform analytical frameworks, improved forensic surveillance systems, standardized toxicological interpretation, and coordinated public health responses.

Overall, addressing the evolving SSC landscape will require sustained multidisciplinary collaboration involving forensic scientists, analytical chemists, clinicians, toxicologists, epidemiologists, and regulatory authorities. Continued advancements in analytical toxicology, surveillance infrastructure, and evidence-based regulation will be essential for improving detection capability, strengthening forensic reliability, and minimizing the public health risks associated with semi-synthetic cannabinoids.

## Figures and Tables

**Table 1:** Summary of major findings from studies investigating semi-synthetic cannabinoids (SSCs) in forensic toxicology, analytical chemistry, pharmacology, and public health.

Research Domain	Research Focus	Major Findings	Key References	Summary of Evidence
**Postmortem and Forensic Toxicology**	Metabolic profiling and biomarker identification	SSCs undergo extensive Phase I and II metabolism, producing hydroxylated, oxidized, and glucuronidated metabolites	[4,14,20,21,22,23]	Parent SSCs are frequently absent in urine; metabolite-focused detection is necessary for reliable interpretation
	Epimer and stereoisomer differentiation	Distinct metabolic and pharmacological profiles exist between 9(R)- and 9(S)-HHC epimers	[5,14,23]	Enantiomer-specific analysis is critical for accurate forensic interpretation and toxicological attribution
	Postmortem uncertainty and toxicological interpretation	Lack of established toxic or lethal concentration ranges complicates causality assessment	[3,4,11,24]	Overlapping metabolites with Δ^9^-THC increase forensic uncertainty, especially in polydrug cases
	Severe intoxication and clinical case reports	SSCs associated with prolonged sedation, neurological impairment, unconsciousness, and cognitive dysfunction	[15,16,17]	Routine toxicology screens often fail to detect SSC exposure without advanced LC–MS/MS or HRMS approaches
	Product mislabeling and adulteration	Commercial products frequently contain undeclared SSCs, impurities, or inaccurate potency labelling	[1,9,18,19]	Mislabeling increases exposure risk and complicates forensic attribution
**Pharmacology and Public Health**	CB1 receptor activation and potency variation	SSCs act as partial CB1 agonists with variable potency depending on structural modification	[2,5,12]	Acetylated and hydrogenated analogues may exhibit altered pharmacodynamic properties
	Regulatory and legal challenges	Many SSCs remain outside conventional drug scheduling systems	[1,3,8,11]	Rapid structural modifications create regulatory loopholes and legal ambiguity
	Youth exposure and vaping-associated risks	SSC-containing vaping products are increasingly marketed as “legal” cannabis alternatives	[6,8,9,10]	Youth exposure and accidental overconsumption are emerging public health concerns
	Neonatal and vulnerable population exposure	Cannabinoids and SSCs detected in authentic breastmilk samples	[13]	Highlights risks for neonates and the need for targeted public health guidance
	Public health surveillance and early warning systems	Seizure analyses and forensic monitoring reveal rapid SSC market expansion	[1,3,7]	Coordinated surveillance systems are required for the timely identification of emerging analogues
**Advanced Analytical Methodologies**	Targeted LC–MS/MS validation	Highly sensitive and validated for biological and commercial matrices	[9,13,23,24,25]	Considered the core analytical platform for routine SSC toxicology
	HRMS/QTOF/Orbitrap methodologies	Enables untargeted detection and structural characterization of novel SSCs	[4,7,10,20,21]	Essential for emerging analogue identification and metabolomics workflows
	Chiral chromatography and epimer differentiation	Chiral separation improves the discrimination of stereoisomers and metabolites	[14,23]	Increasingly important for HHC-related forensic investigations
	GC–MS confirmatory applications	Useful for seized material characterization but limited by thermal instability	[7,11,17]	Often used as a supplementary analytical approach
	Multi-platform and harmonized workflows	Combined targeted and untargeted strategies improve forensic reliability	[3,7]	Integrated analytical frameworks support standardized toxicological interpretation

**Table 2:** Comparative overview of advanced analytical techniques used for semi-synthetic cannabinoid detection and characterization.

Technique	Major Strengths	Main Limitations	Major Applications in SSC Analysis	Representative References
LC–MS/MS	High sensitivity and specificity; excellent quantitative performance; suitable for thermolabile compounds; validated across multiple biological matrices	Limited differentiation of structural isomers and epimers without advanced chromatographic separation	Routine targeted detection and quantification of SSCs and metabolites in blood, urine, breastmilk, vaping liquids, and edible products	[4,9,13,23,24,25]
LC–HRMS/MS (Orbitrap/QTOF)	Accurate mass determination; non-targeted screening capability; metabolite discovery; structural elucidation	Expensive instrumentation; complex spectral interpretation; limited stereochemical discrimination	Identification of novel SSCs, metabolite profiling, untargeted toxicological screening, seizure analysis	[3,4,7,10,20,21]
UHPLC–QTOF-MS	High chromatographic resolution; rapid analysis; effective metabolomics workflow	Requires advanced expertise and spectral libraries; data processing complexity	Biomarker discovery, urine metabolite characterization, and forensic screening	[14,20,21]
GC–MS	Widely available; robust spectral databases; effective confirmatory technique	Thermal degradation of SSCs; derivatization often required; poor suitability for thermolabile compounds	Preliminary screening and confirmatory analysis of seized materials and commercial products	[7,11,17]
Chiral LC and Epimer-Specific Chromatography	Differentiates stereoisomers and enantiomers; improves interpretation accuracy	Limited availability of validated protocols and reference standards	Resolution of 9(R)- and 9(S)-HHC epimers; forensic interpretation of enantiomer-specific metabolism	[5,14,23]
Metabolomics-Based Approaches	Detects exposure through metabolite patterns even when parent compounds are absent	Requires HRMS infrastructure and advanced bioinformatics	Biomarker discovery and retrospective exposure assessment	[20,21,22]
Multi-Platform Analytical Strategies	Combines the strengths of targeted and untargeted approaches; improves reliability	Increased cost and analytical complexity	Comprehensive forensic workflows integrating LC–MS/MS, HRMS, and GC–MS	[3,7,17]

## Data Availability

Not applicable.

## Abbreviations

**Abbreviation**	**Full Term**
SSCs	Semi-Synthetic Cannabinoids
CBD	Cannabidiol
Δ^8^-THC	Delta-8-Tetrahydrocannabinol
Δ^9^-THC	Delta-9-Tetrahydrocannabinol
Δ^10^-THC	Delta-10-Tetrahydrocannabinol
HHC	Hexahydrocannabinol
HHCP	Hexahydrocannabiphorol
HHC-O	HHC-O-Acetate
HHC-P	Hexahydrocannabinol-P
HHC-C8	Hexahydrocannabinol-C8 Analogue
HHC-C9	Hexahydrocannabinol-C9 Analogue
H4-CBD	Hexahydrocannabidiol
THCP	Tetrahydrocannabiphorol
CB1	Cannabinoid Receptor Type 1
CB2	Cannabinoid Receptor Type 2
SCRA	Synthetic Cannabinoid Receptor Agonist
LC–MS/MS	Liquid Chromatography–Tandem Mass Spectrometry
LC-HRMS	Liquid Chromatography–High-Resolution Mass Spectrometry
HRMS	High-Resolution Mass Spectrometry
GC–MS	Gas Chromatography–Mass Spectrometry
UHPLC	Ultra-High-Performance Liquid Chromatography
UHPLC-QTOF-MS	Ultra-High-Performance Liquid Chromatography–Quadrupole Time-of-Flight Mass Spectrometry
QTOF	Quadrupole Time-of-Flight
LC-QTOF-MS	Liquid Chromatography–Quadrupole Time-of-Flight Mass Spectrometry
Orbitrap	Orbitrap High-Resolution Mass Spectrometer
MRM	Multiple Reaction Monitoring
LC	Liquid Chromatography
GC	Gas Chromatography
MS	Mass Spectrometry
Phase I	Phase I Metabolism
Phase II	Phase II Metabolism
QDF	Qualitative Data Framework
